# SARS‐CoV‐2 does not infect pigs, but this has to be verified regularly

**DOI:** 10.1111/xen.12772

**Published:** 2022-08-30

**Authors:** Tanja Opriessnig, Yao‐Wei Huang

**Affiliations:** ^1^ The Roslin Institute and The Royal (Dick) School of Veterinary Studies University of Edinburgh Easter Bush Midlothian UK; ^2^ Department of Veterinary Diagnostic and Production Animal Medicine College of Veterinary Medicine Iowa State University Ames Iowa USA; ^3^ Guangdong Laboratory for Lingnan Modern Agriculture College of Veterinary Medicine South China Agricultural University Guangzhou China

**Keywords:** COVID‐19, cross‐species transmission, pig models, SARS‐CoV‐2, therapeutics, xenotransplantation

## Abstract

For successful xenotransplantation, freedom of the xenocraft donor from certain viral infections that may harm the organ recipient is important. A novel human coronavirus (CoV) with a respiratory tropism, designated as SARS‐CoV‐2, was first identified in January 2020 in China, but likely has been circulating unnoticed for some time before. Since then, this virus has reached most inhabited areas, resulting in a major global pandemic which is still ongoing. Due to a high number of subclinical infections, re‐infections, geographic differences in diagnostic tests used, and differences in result reporting programs, the percentage of the population infected with SARS‐CoV‐2 at least once has been challenging to estimate. With continuous ongoing infections in people and an overall high viral load, it makes sense to look into possible viral spillover events in pets and farm animals, who are often in close contact with humans. The pig is currently the main species considered for xenotransplantation and hence there is interest to know if pigs can become infected with SARS‐CoV‐2 and if so what the infection dynamics may look like. This review article summarizes the latest research findings on this topic. It would appear that pigs can currently be considered a low risk species, and hence do not pose an immediate risk to the human population or xenotransplantation recipients per se. Monitoring the ever‐changing SARS‐CoV‐2 variants appears important to recognize immediately should this change in the future.

## 1 INTRODUCTION

The ongoing COVID‐19 pandemic associated with SARS‐CoV‐2, was initially observed in a cluster of patients with severe respiratory disease in Wuhan, Hubei Province, China during December 2019.^1^ The causative agent, a novel coronavirus (CoV), initially called 2019‐nCoV but later re‐named to SARS‐CoV‐2, was identified and reported to the World Health Organization (WHO) on January 9, 2020.^1^ On March 11, 2020 the WHO upgraded the SARS‐CoV‐2 outbreak to global pandemic status.^2^ Overall the infection pattern of SARS‐CoV‐2 in the global human population has been remarkable. A pool of approximately 7.753 billion SARS‐CoV‐2 naïve people were present when the virus started infecting humans. Based on data from the United States and eight European countries including France, Italy, Spain, Germany, Belgium, Switzerland, Netherlands, and the United Kingdom, it has been determined that in the early phase of the pandemic, SARS‐CoV‐2 grew exponentially at rates between 0.18 and 0.29 per day which correlates to epidemic doubling times between 2.4 and 3.9 days.^3^ The virus spread rapidly through most countries and continents resulting in a high number of infected people shedding SARS‐CoV‐2 for extended periods of time. A recent study estimated that by November 14, 2021 over 40% of the global population had been infected with SARS‐CoV‐2 at least once.^4^

## 2 ANIMALS IN WHICH NATURAL OR EXPERIMENTAL SARS‐CoV‐2 INFECTION HAS BEEN CONFIRMED

**Pets**. As people naturally have close relationships with their pets, it comes as no surprise that human‐to‐cat transmission was reported on March 28, 2020 in a cat from a Belgian COVID‐19 household.^5^ This first documented spillover of SARS‐CoV‐2 into animals was quickly followed by detection of the virus in additional cats,^6^, ^7^, ^8^ and also in dogs.^9^, ^10^, ^11^ Very recently a suspected cat‐to‐human SARS‐CoV‐2 transmission was documented for the first time.^12^ Specifically, a SARS‐CoV‐2 positive cat from a COVID‐19 household sneezed into the face of a veterinarian while it was being examined, and it is hypothesized that the veterinarian was infected by the cat which was previously infected by the owner.^12^ Moreover, naturally SARS‐CoV‐2 infected Syrian hamsters in a Hong Kong pet shop have been associated with multiple events of hamster‐to‐human infection by the Delta variant of SARS‐CoV‐2, further resulting in a sustained human‐to‐human transmission.^13^ Experimentally infected rabbits have also been shown to shed infectious SARS‐CoV‐2 from the nose and throat.^14^ At the time of writing, SARS‐CoV‐2 infection has been confirmed in other companion animals including golden hamsters^15^ and ferrets.^16^ Interestingly, guinea pigs appear to be resistant to the virus.^17^ In contrast, almost 20 years ago when SARS‐CoV‐1 appeared, guinea pigs were experimentally infected with reovirus alone or concurrent SARS‐CoV‐1, both isolated from a human case.^18^ Guinea pigs infected with reovirus alone died between 22 and 30 days after experimental infection while co‐infected guinea pigs died between 4 and 7 days and expressed similar gross lung lesions as described in human patients, highlighting the possible importance of coinfections in disease expression.^18^

**Farmed animals/livestock**. The first cases of natural SARS‐CoV‐2 in farmed animals were discovered on two commercial mink farms in the Netherlands in April 2020.^19^ The mink had developed respiratory symptoms with increased mortality, and it was determined that 68% of the workers on farm had also evidence of SARS‐CoV‐2 infection. Moreover, besides human‐to‐mink transmission, mink‐to‐human transmission could also be confirmed.^19^ Experimental infection of cattle with SARS‐CoV‐2 resulted in low levels of virus replication and antibody development; however, transmission to non‐infected controls did not occur.^20^ In a similar study, cattle, sheep, goats, alpacas, and horses were infected intranasally with SARS‐CoV‐2.^21^ Virus isolation was successful in one of three cattle; however, neutralizing antibody levels were absent or low in most animals after 1 month, suggesting a minor role of cattle in SARS‐CoV‐2 epidemiology.^21^ In a different experimental study, sheep‐derived cell cultures supported SARS‐CoV‐2 replication, but experimental infection of sheep resulted in low viral RNA levels in nasal and oral swabs right after challenge, and in the respiratory tract and lymphoid tissues 4 and 8 days later, indicating a low susceptibility of sheep to SARS‐CoV‐2 infection.^22^ Experimentally infected poultry (chickens, ducks, and turkeys) were found to be resistant to SARS‐CoV‐2 infection.^23^ Due to their importance in xenotransplantation pigs are discussed in more detail in Section 6.

**Zoo animals and other wildlife**. Several zoo animals have been found to be SARS‐CoV‐2 positive, thought to be infected by transmission from care staff or zoo visitors. Affected animals include lions and tigers,^24^ snow leopards, otters, non‐human primates, gorillas, a binturong, a fishing cat, a coatimundi, hyenas, hippopotamuses, and manatees.^25^, ^26^ SARS‐CoV‐2 infection has also been identified in other wildlife, including white‐tailed deer,^27^ a mule deer,^28^ a black‐tailed marmoset,^29^ a giant anteater,^30^ wild otters,^31^ wild mink,^32^ fruit bats,^33^ and racoon dogs,^34^ which in the last two cases was experimentally confirmed. Tree shrews have been shown to develop subclinical infection after experimental infection,^35^ and bank voles could be experimentally infected with SARS‐CoV‐2 but there was no transmission to contact controls.^36^ A study of SARS‐CoV‐2 antibodies in samples from wild jackals, red foxes, and yellow legged gulls in Croatia collected during June–November 2020, assessed by a commercial N‐protein‐based double antigen enzyme‐linked immunosorbent assay (ELISA; ID Screen SARS‐CoV‐2 Double Antigen Multi‐species ELISA; ID.Vet, France), demonstrated that 4.6% (3/65) of jackals, 2.9% (6/204) of red foxes and none of yellow legged gulls (0/111) were positive for SARS‐CoV‐2 antibody.^37^ A confirmatory surrogate virus neutralization test based on the S‐gene (GenScript, Netherlands) could not confirm the positive ELISA results and all animals were also negative for presence of SARS‐CoV‐2 RNA in fecal samples.^37^ When using serology for assessing possible infections it is important to understand that the response could be specific and true, however, there could also be cross‐reaction to other circulating CoVs in addition to non‐specific binding. Caution is needed when using serology assays validated on few SARS‐CoV‐2 positive species, and tested in animals where positive controls are not available. The double antigen ELISA described above has been validated on serum, plasma and whole blood and the species range is described by the manufacturer as minks, ferrets, dogs, cattle, sheep, goats, horses, and other susceptible species. In the above study blood was collected from wild boars and yellow‐legged gulls, while for all other investigated animal species muscle extracts from carcasses were collected and used, which is not a validated sample.^37^

Among the cases of SARS‐CoV‐2 identified in wildlife, those in wild white‐tailed deer, raised initial concerns due to the high prevalence rates of SARS‐CoV‐2 in deer and the presence of white‐tailed deer in suburban areas close to humans.^27^ Based on available data, free‐ranging white‐tailed deer appear highly susceptible to infection with SARS‐CoV‐2 and have been exposed to multiple SARS‐CoV‐2 variants from humans.^27^, ^38^, ^39^, ^40^ Furthermore, white‐tailed deer are capable of sustaining transmission in the wild population. Importantly, clinical disease has not been observed in free‐ranging white‐tailed deer but virus and antibodies have been confirmed in wild populations across the United States.^27^, ^38^, ^39^, ^40^ Experimental infection of white‐tailed deer further confirmed that this species is susceptible to SARS‐CoV‐2, and the virus was subsequently transmitted to uninfected deer comingled with the infected animals.^41^ In addition to deer, North American deer mice were also recently found to be susceptible to SARS‐CoV‐2 after experimental infection.^42^ Infected deer mice experienced subclinical disease but shed infectious virus via nasal sections, feces, and urine.^42^ As these deer mice do not tend to live in urban or suburban areas, they may not pose an additional risk to humans.

With such a wide range of possible SARS‐CoV‐2 animal reservoirs, the potential for infection of immunosuppressed transplant patients around the time of transplantation, or more specifically via pig tissues or organs through xenotransplantation, is a concern.

## 3 SARS‐CoV‐2 VARIANTS CURRENTLY RECOGNIZED

Since its introduction into humans, SARS‐CoV‐2 has continually evolved. Different labels to describe virus strains have been used, including lineage (i.e., a group of closely related viruses with a common ancestor), and variant (i.e., a viral genome that may contain one or more mutations).^43^ The following variant categories are commonly used:

1. *Variants of concern (VOC)*: This describes variants that have produced a significant difference in transmissibility, disease severity or interference with the immune system, or a combination of these, which may impact the epidemiological situation. Current VOC include viruses from the Delta (B.1.617.2) and Omicron lineages (BA.1, BA.2, BA.4 and BA.5).
2. *Variants of interest (VOI)*. For these variants, genomic, epidemiological or in‐vitro evidence is available indicating a potential impact on transmissibility, severity and immunity or any combination, with a high potential to affect the epidemiological situation. No variants currently fall into this category.
3. *Variants under monitoring (VUM) or being monitored (VBM)*. This describes SARS‐CoV‐2 variants that have been detected through epidemic intelligence, rules‐based genomic variant screening, or for which preliminary scientific evidence exists. It is possible that these variants have properties similar to those of a VOC, but the evidence is weak or has not yet been assessed. At least one outbreak must have been reported for a variant to fall into this category.
4. *De‐escalated variants*. This group includes variants that are no longer circulating and never had any impact or concerning properties.

Other criteria used to differentiate SARS‐CoV‐2 virus groups include the WHO label (for example: Alpha, Beta, Delta, Omicron), lineage, year and month of discovery, country of discovery, and evidence concerning properties such as transmissibility, immunity or infection severity, or additional mutations such as those described for the Pango lineages and any other characteristic protein changes. Mutations of interest include spike protein mutations such as for example amino acids (aa) 319–541, the receptor binding domain (RBD), and the S1 part of the S1/S2 junction and a small stretch on the S2 side (aa 613–705).^43^

A study investigating lineage diversity of SARS‐CoV‐2 from human and non‐human hosts found 28 lineages in non‐human hosts.^44^ The majority was represented by Delta (n = 1105, 30.7%) and Alpha variants (n = 466, 12.9%), followed by a Danish B.1.1.298 lineage (n = 458, 12.7%). Non‐human hosts with the high numbers of lineages included American mink with 14 lineages, white‐tailed deer with 12 lineages, and the domestic cat with 10 lineages. The study authors proposed that interspecies transmission of SARS‐CoV‐2 between animals and humans could result in novel variants evolving within an animal reservoir, with potential to spill back into humans, and this should be considered in order to establish better pathogen surveillance and containment strategies.^44^ The potential for evolution of novel variants in animal species, and spillover back into humans, is an additional risk associated with xenotransplantation.

## 4 SARS‐CoV‐2 ORIGIN

The origin of SARS‐CoV‐2 is still highly controversial, with debate focusing on two competing ideas: a zoonotic emergent event or a laboratory escape from the Wuhan Institute of Virology (WIV).^45^ Despite extensive contact tracing of early COVID‐19 cases, there is no evidence of an epidemiological link with WIV staff during the early stages of the outbreak. WIV staff, and particularly the bat CoV research group at the center of this debate, were also all found to be serologically negative for SARS‐CoV‐2 antibodies in March 2020, and SARS‐CoV‐2 or closely related viruses were not included in the extensive catalogue of viruses held at the WIV. In addtion, propagation of SARS‐CoV‐2 or a progenitor virus using the standard methodology of the time consistently results in loss of the furin cleavage site, which is an important feature of SARS‐CoV‐2, and this loss was not identified in early circulating strains. Furthermore, based on many subsequent infection studies, transmission of SARS‐CoV‐2 into common laboratory animals is challenging, making it unlikely that laboratory animals could have served as initial reservoir. Zoonotic transmission from an unknown animal reservoir, as was the case for previous CoV outbreaks, appears far more likely.^45^ It has been suggested that SARS‐CoV‐2 passaged in several animal species before entering humans. The SARS‐CoV‐2 genome was found to be 96.2% identical to the bat CoV RaTG13.^46^ However, it appears a significant evolutionary gap exists between SARS‐CoV‐2 and the closest related known animal viruses: for example, the bat virus RaTG13 has a genetic distance of approximately 4% corresponding to roughly 1150 mutations from the Wuhan‐Hu‐1 reference sequence for SARS‐CoV‐2, reflecting decades of evolutionary divergence.^45^ Similarly, the Malayan pangolin, for some time favored to be an important link in the SARS‐CoV‐2 evolution,^47^, ^48^ can likely be ruled at this point.^45^ It appears that the intermediate animal species responsible for the outbreak in humans has not yet been identified, and will most likely remain undetected as the prevalence of any progenitor virus with no clinical signs may be low.^45^ Very recently two studies were published supporting that the Huanan Seafood Wholesale Market in Wuhan, China was likely the early epicenter of transmission of SARS‐CoV‐2 into people.^49^ The authors of the first study demonstrated that 55 of 168 of the earliest known COVID‐19 cases were clustered in close proximity to the market.^49^ The second study looked at SARS‐CoV‐2 sequences obtained early in the pandemic,^50^ and only two distinct lineages were observed which, based on further analysis, were the result of at least two separate cross‐species transmission events into humans close together, estimated to have occurred during October through December 2019. The authors point out that the window between SARS‐CoV‐2 first jumping into humans and first cases of COVID‐19 being reported was very narrow.^50^

## 5 SARS‐CoV‐2 RECEPTOR BINDING

A requirement for successful replication in both humans and non‐human species, including pets and farm animals, is having a suitable angiotensin‐converting enzyme 2(ACE2) which serves as a functional receptor for the spike protein of SARS‐CoV‐2.^51^ The ACE2 receptor is widely distributed in animals, particularly in the cardiovascular system and in alveolar epithelial cells.^52^ It has been shown that adaptive viral genome mutations can potentially change the virulence of the virus.^53^ For example, a single amino acid exchange can reduce or enhance the ability of the virus to bind a particular ACE2 receptor or evade the immune system, which complicates SARS‐CoV‐2 spike protein‐based vaccine development.^53^ The spike protein RBD is therefore essential to both successful infection and vaccine escape. The main SARS‐CoV‐2 strains, categorized initially into Alpha, Beta, Delta, Gamma, and Omicron, all have mutations in the spike protein RBD and the N‐terminal domain. The N501Y mutation, located on the RBD, is common to all variants except the Delta variant, and triggers increased affinity of the spike protein to the ACE2 receptors, thereby enhancing the viral attachment and its subsequent entry into the host cells.^53^ Once the importance of ACE2 in human SARS‐CoV‐2 infection was realized, numerous studies have focused on identifying animal species that have ACE2 receptors similar enough to humans to become a potential reservoir for the virus.^51^ In silico structural homology modelling, protein–protein docking, and molecular dynamics simulation studies of the SARS‐CoV‐2 spike protein's ability to bind ACE2 from relevant species indicate that the highest binding affinity is to human ACE2, then to pangolin ACE2, whereas the affinity to monkey ACE2 was much lower.^54^ Interestingly, species with a high predicted ACE2 binding affinity range, for example monkeys, hamsters, dogs, ferrets, and cats, have all been reported to be permissive to SARS‐CoV‐2 infection based on experimental data or natural infection. These findings would support a correlation between binding affinity and infection susceptibility.^54^ Other similar studies confirmed these results and also predicted that the ACE2 receptor from other animals such as dogs, tigers, camels, cats, dwarf hamsters, and sheep may have a slightly increased affinity to the SARS‐CoV‐2 RBD.^55^

## 6 PIGS AND SARS‐CoV‐2

Whether or not SARS‐CoV‐2 can infect domestic pigs is of particular concern, not only because these animals are economically important and raised under intensive farming conditions, but also because this species is also being used in xenotransplantation. Pigs are the most commonly used species in xenotransplantation due to their ready availability and the similarity of their organs to human organs. During xenotransplantation the xenograft recipient is usually immunosuppressed, and hence very susceptible to any viral infection that is introduced from the organ donor species. For example, the first human patient to receive a pig heart in January 2022^56^ died 8 weeks after the successful surgery due to a porcine cytomegalovirus infection, a common herpes virus in pigs.^57^ It is well known that there are certain risk factors for patients developing COVID‐19 and requiring re‐admittance to hospital, which include solid organ transplantation.^58^ When liver transplant recipients were assessed following vaccination for SARS‐CoV‐2, they were found to have a considerably lower immunological response to the Pfizer‐BioNTech SARS‐CoV‐2 mRNA‐based vaccine^59^ compared to normal healthy people. Factors predicting a reduced vaccine response also include older age, reduced renal function and immunosuppressive medications.^59^ If a virus such as SARS‐CoV‐2 was introduced during xenotransplantation, this would obviously be detrimental for the patient. Investigations to establish whether or not pigs can support and sustain SARS‐CoV‐2 infections are therefore of great importance. The work done so far can be divided into studies where pigs were experimentally infected with SARS‐CoV‐2, studies assessing the ability of pig cells lines to support SARS‐CoV‐2 replication, and studies determining virus prevalence data from the field.

1. **In vivo pig studies**. Several studies investigated the ability of SARS‐CoV‐2 to infect and replicate in pigs (Table 1). While some results are conflicting,^60^ the body of evidence suggests that pigs are not susceptible to SARS‐CoV‐2.^33^, ^61^, ^62^, ^63^, ^64^, ^65^ SARS‐CoV‐2 infected pigs typically do not develop any clinical signs or lesions, but can mount an antibody response after parenteral virus administration.^62^, ^63^, ^64^, ^65^ Viral RNA can be found for up to 1–3 days after inoculation, likely suggesting inoculum remnants.^62^, ^63^, ^64^, ^65^ SARS‐CoV‐2 RNA was also found in lung tissue of some pigs on days 3, 6, and 9, without evidence of virus replication.^64^ A single study reported successful SARS‐CoV‐2 virus isolation from a lymph node 13 days after experimental infection using the intranasal inoculation route.^60^ Transmission of SARS‐CoV‐2 from infected to contact pigs did not occur in any of the studies published so far.^33^, ^60^, ^61^, ^62^, ^63^, ^64^, ^65^
2. **In vitro studies using porcine cells**. SARS‐CoV‐2 studies using cell lines of porcine origin are summarized in Table 2. It appears that porcine kidney (PK) 15 cells can be readily infected with SARS‐CoV‐2,^33^, ^66^ often resulting in cytopathogenic effects (CPE) suggestive of virus‐related structural changes in the host cells.^62^ Cellular restriction factors are important to prevent certain viral infections.^67^, ^68^ It should be underlined that PK15 cells have lost certain intracellular restriction factors and therefore may be susceptible to not only SARS‐CoV‐2 but also other viruses, for example porcine endogenous retroviruses (PERV).^69^ A similar study also reported an increase in SARS‐CoV‐2 genomic copy number in swine kidney (SK) 6 cells,^33^ and a study using swine testicle (ST) cells also resulted in virus increase and visible CPE.^62^ However, as SARS‐CoV‐2 is a respiratory virus which mainly replicates in the upper respiratory tract and lungs, the importance of successful virus replication in individual cell lines derived from kidneys or testicles is questionable. One recent study infected primary cell cultures from the respiratory tract of various animal species, including pigs, with SARS‐CoV‐2.^70^ Another infected nasal mucosa explants, tracheal epithelial cells cultured at an air‐liquid interface and precision cut lung slices from pigs.^66^ While productive replication of SARS‐CoV‐2 was observed in dogs and few other species, none of the pig tissues used supported viral replication.^66^, ^70^ Enhanced apoptosis in primary respiratory epithelial cells has been considered as a possible mechanism to self‐limit SARS‐CoV‐2 replication in the pig respiratory tract.^71^ In addition, it may be interesting to know whether the cell lines used for SARS‐CoV‐2 infection studies were infected with other viruses, for example a porcine circovirus (PCV) 1, which was found to be a contaminant in cells used for production of a human rotavirus vaccine during 2010.^72^, ^73^
3. **Surveillance studies**. From June to December 2020 blood, muscle extract and fecal samples from 153 free‐living wild boars in Croatia were tested and it was found that 6/153 blood samples (3.9%) were positive by a commercial multispecies SARS‐CoV‐2 antibody ELISA, whereas all fecal samples were negative for SARS‐CoV‐2 RNA by PCR testing.^37^ As outlined in Section 2 under zoo animals and other wildlife, the specificity of the obtained responses should be confirmed to rule out cross‐reactions to other CoVs and non‐specific binding. In the Netherlands, 417 pig serum samples were obtained at slaughter from 17 farms located in a region with a high human case incidence of SARS‐CoV‐2 from March to July 2020.^64^ These samples were tested with protein micro array, plaque reduction neutralization test (PRNT) and receptor‐binding‐domain ELISA. None of the serum samples were positive in all three assays, although six samples from one farm returned a low positive result in PRNT (titers 40–80). In addition, the authors also investigated an outbreak of respiratory disease in pigs on one farm, which occurred in February 2021, coinciding with recent exposure to SARS‐CoV‐2 infected animal caretakers. Tonsil swabs and paired serum samples were tested and there was no evidence for infection with SARS‐Co‐2. The authors concluded that sporadic infections in the field cannot be excluded, but large‐scale SARS‐CoV‐2 transmission among pigs is unlikely.^64^

**TABLE 1:** Summary of in vivo SARS‐CoV‐2 infection studies using pigs. Table adapted from Sikkema R.S. et al.^64^

			Inoculation						Outcomes			
Pig breed/age at infection	Infected pigs #	Contact pigs #	*Route*	*Dose*	*Volume*	Day(s) of necropsy	Source farm health status	Concurrent infections	*RNA shedding*	*Virus replication*	*Serum antibody development*	References
German Landrace 9 weeks	9	3 (24 h)^a^	IN	10^5^ TCID_50_	NA	4, 8, 12, 21	SPF^b^	Not described	ND	ND	ND	^33^
Landrace x Large White 6 weeks	5	3 (0 h)	IN	10^4.5^ PFU	NA	14	SPF	Not described	ND	ND	ND	^61^
Breed not described 5 weeks	9	6 (0 h)	Oral, IN and IT	10^6^ TCID_50_	4 ml	4, 8, 21	SPF	Free of porcine circovirus type 2 (PCV2), swine influenza virus (SIV) and porcine reproductive and respiratory syndrome virus (PRRSV)	ND	ND	ND	^62^
Landrace x Large White 5‐6 weeks	5	None	IN	10^5.8^ TCID_50_	3 ml	1, 2	Conventional	Seropositive for porcine respiratory coronavirus (PRCV). Not described for other pathogens	D1: 1/5 trachea swabs	ND	ND	^65^
	5	None	IT		3 ml	2, 22			ND	ND	D22: 5/5 N antibodies and VN titers	
	5	None	IM		2 ml	2,22			ND	ND	D14 and D22: 5/5 spike antibodies D22: 5/5 VN titers	
	5	None	IV		2 ml	2, 22			ND	ND	D14 and D22: 5/5 spike antibodies D22: 5/5 VN titers	
American Yorkshire crossbred 8 weeks	16	2 (10 days)	Oral, IN	1×10^6^ PFU	3 ml	3, 5, 7, 9, 11, 13, 15, 22 and 29	SPF	Not described	D3: 1/2 oral fluids D3: 2/16 nasal washes	D13: Sucessful virus isolation from a lymph node	D6: 1/2 oral fluid D11: 2/8 pigs VN titers D13: 2/6 pigs VN titers	^60^
Large White or Large White x Norsvin Landrace 12 weeks	8	None	IN and IT	10^6.2^ TCID_50_	10 ml	3, 6, 10, 21	Conventional	Not described	D1: 8/8 and D2: 1/8 oropharyngeal swabs D1: 2/8 nasal swabs	ND	D10: 2/4 pigs VN titers D14: 1/1 previous positive pig (the other positive pig was euthanized on D10)	^64^
Breed not described 3 weeks	4	1 (2 days)	IV	6.8×10^6^ TCID_50_	2 ml	21	SPF	Not described	D4: 1/4 oranasal swab D3: 2/4 buffy coat	ND	D7: 4/4 VN titers D14: 2/4 VN titers (reduced) D 21: 0/4 VN titers	^63^
	4	1 (2 days)	IT		5 ml	21			D1: 4/4 D2: 2/4 D3‐7: 1/4 All oronasal swabs	ND	D14: 1/4 and D21: 1/4 (different pig each day) VN titers	
	4	1 (2 days)	IN		5 ml	21			D1: 4/4 D2: 2/4 D3: 1/4 All oronasal swabs	ND	D14 and D21: 1/4 VN titers	

Abbreviations: D, day post SARS‐CoV‐2 challenge; IM, intramuscular; IN, intranasal; IT, intratracheal; IV, intravenous; N, nucleocapsid protein; NA, not applicable; ND, not detected; PFU, plaque forming unit; TCID_50_, 50% tissue culture infectious dose; VN, titers determined via a virus neutralization assay.

^a^ Time of introduction of contact pigs after infection of infected pigs.

^b^ SPF, specific pathogen free (SPF). The pig herd is free of certain economically important pig pathogens and is monitored at regular intervals to maintain this status. SPF for the purpose of this table also includes pigs designated as “high health” in contrast to the term “conventional pig farm.” A conventional pig farm is not claiming to be free of one or more specified pathogens, likely has a number of endemic disease pathogens present, but is not required to follow regular disease monitoring programs.

**TABLE 2:** Summary of in vitro SARS‐CoV‐2infection studies using pig derived cell lines

Cell line	SARS‐CoV‐2 dose	Passages or hpi	Results	Cytopathogenic effects (CPE)	Tests for other viruses	Reference
Porcine kidney (PK) 15	10^5.5^ TCID_50_	2 and 72 hpi	Ct 27.7 and 24.2 Titer: negative	No	ND	^33^
	0.05 MOI of passage 3 of the VeroE6‐passaged SARS‐CoV‐2	4 passages		CPE at passage 4	ND	^62^
	0.1 MOI for 2 h at 37°C	2, 24, 72 and 120 hpi	3 log or greater increase in mean viral load over a period of 120 h	No	ND	^66^
Swine kidney (SK) 6	10^5.5^ TCID_50_	27.1	Ct 27.1 → 12.0 Titer: 6.8×10^7^	No	ND	^33^
Swine testicle (ST) cells	10^5.5^ TCID_50_	27.8	Ct 27.8 → 11.2 Titer: 3.1×10^6^	No	ND	^33^
	0.05 MOI of passage 3 of the VeroE6‐passaged SARS‐CoV‐2	2 passages		CPE at passage 2	ND	^62^
Primary porcine respiratory epithelial cells	MOI 5.0, 5.0 × 10−2 5.0 × 10−4	120 hpi	Ct 17.5 with MOI 5.0.	CPE dose and time dependent, most prominent with MOI 5.0 infectious dose and 96 hpi	ND	^71^

Abbreviations: C_t_, cycle threshold; hpi, hours post infection; MOI, multiplicity of infection; ND, not described; TCID_50_, 50% tissue culture infectious dose.

## 7 DEVELOPMENT OF A HUMANIZED SWINE MODEL FOR COVID‐19 RESEARCH

For a virus infection that primarily targets humans, such as SARS‐CoV‐2, animal models that accurately mimic human disease manifestations and can be used as models for vaccine and therapeutics development, are incredibly important. A variety of animal SARS‐CoV‐2 models are currently available, including hamsters,^74^, ^75^ mice,^76^, ^77^ ferrets,^16^ cats,^78^ and non‐human primates.^79^, ^80^ However, from the perspectives of both ethics and availability, usage of non‐human primates should be limited whenever possible, while ferrets, mice, hamsters, and cats simply do not resemble human infections enough to be useful for testing intervention treatments. In contrast, pigs closely resemble human disease processes in general, because their anatomy and physiology are similar. While current data suggest that the ordinary domestic pig is not susceptible to SARS‐CoV‐2, recent research has been focusing on modifying pigs to change this. To create a COVID‐19 pig model, one research group inserted the human ACE2 (hACE2) receptor at the pig ACE2 locus using the CRISPR/Cas9 system to create hACE2 knock‐in pigs.^81^ To determine the replication efficiency of SARS‐CoV‐2 in primary cells derived from these hACE2 knock‐in pigs, lung and kidney epithelial cells were isolated and infected with SARS‐CoV‐2 at a MOI of 0.01. In contrast to cells from wild‐type pigs, these primary hACE2 knock‐in pig cells showed significant CPE after 72 h of infection.^81^ The results of further in vivo studies of SARS‐CoV‐2 infection in these pigs will hopefully be available soon.

## 8 PRODUCTION OF SWINE GLYCO‐HUMANIZED POLYCLONAL ANTIBODY AS THERAPY FOR COVID PATIENTS

During the early stages of the pandemic few treatment options for severely impacted or immunocompromised patients were available, and passive antibody therapy using convalescent plasma was explored as a treatment for COVID‐19. However, the initial reports of therapeutic benefits in immunocompetent patients were not supported in larger studies, possibly because the antibody quality and quantity could not be standardized, and antibodies raised against a certain SARS‐CoV‐2 variant may not always fully protect against other variants.^82^, ^83^ As an alternative, the swine glyco‐humanized polyclonal antibody XAV‐19 was developed using alpha 1,3‐galactosyltransferase (GGTA1) and cytidine monophosphate N‐acetylneuraminic acid hydroxylase (CMAH) gene double knockout pigs immunized against SARS‐CoV‐2 spike RBD.^84^ XAV‐19 targets epitopes distributed all over the RBD, and particularly the receptor binding motif. In a subsequent spike/ACE‐2 interaction assay, XAV‐19 showed potent neutralization capacities against the original Wuhan, United Kingdom (Alpha/B.1.1.7) and South African (Beta/B.1.351) variants. These results were confirmed by cytopathogenic assays using Vero E6 cells and live virus variants including the Gamma/P.1 and the Delta/ B.1.617.2 variants.^85^

## 9 ANIMAL MODELS FOR STUDYING SARS‐CoV‐2 OMICRON AND OTHER VARIANTS

At the beginning of the pandemic there was an immediate need to learn more about SARS‐CoV‐2 biology, transmission, evolution, and prevention. Due to a lack of knowledge about the virus, and the availability of large research funds almost instantly, a number of studies were conducted during 2020 and 2021. Only at the end of 2020, when many studies were already ongoing or finished, the emergence of different SARS‐CoV‐2 variants was recognized, which potentially differed in their capacity to cause disease, their transmissibility, or their availability to evade immune responses. To date there is little information on these novel SARS‐CoV‐2 variants in animal models. Results of studies using transgenic mice carrying human ACE2 indicate that, compared to the Alpha, Beta, and Delta variants, Omicron appears to be milder in virulence, based on insignificant weight loss and a low mortality in Omicron infected mice.^86^, ^87^, ^88^ In the hamster model, Alpha and Beta variants were both able to replicate efficiently, and viral infection resulted in bronchopneumonia similar to that observed in COVID‐19 patients. However, there was no difference between these two SARS‐CoV‐2 variants.^89^ When the Delta and Omicron variants were also compared in this model, infection with the Delta variant resulted in quicker weight loss and more rapid distribution of SARS‐CoV‐2 protein in the lungs, resulting in increased pathogenicity.^90^ In ferret models, it was found that both the prototype Wuhan strain and the D614G variant can infect ferrets, but the D614G variant was dominant in terms of progeny virus produced from these animals during dual infection.^91^ Non‐human primates have also been infected with Beta, Gamma, and Delta variants to assess vaccine efficacy against different variants.^92^ Established nomenclature systems for naming and tracking SARS‐CoV‐2 genetic lineages by GISAID, Nextstrain and Pango are currently and will remain in use by scientists and in scientific research.^93^ However, the current system is complicated and, due to different classification schemes of SARS‐CoV‐2, strategies should be developed to characterize lineages/variants/mutants in a more controlled fashion. A single common classification system of strains should perhaps be implemented so that results in publications can be better compared by people not familiar with virus sequence comparisons. For pigs, to date challenge studies have been done with the following isolates: 2019_nCoV Muc‐IMB‐1,^33^ CTan‐H,^61^ hCoV‐19/USA/WA1/2020 (GISAID ID EPI_ISL_404895) USA‐WA1/2020 isolate (GenBank accession # MN985325),^62^ GISAID ID EPI_ISL_510689,^65^ and hCoV‐19/Canada/ON‐VIDO‐01/2020 (GISAID accession no. EPI_ISL_425177).^60^ All these strains appear to have evolved from the Wuhan SARS‐CoV‐2 isolate. In addition, SARS‐CoV‐2/human/NL/Lelystad/2020 (wild‐type D614G, Genbank accession number MZ144583),^64^ and TGR/NY/20 (wild‐type D614G, GenBank accession number MT704317), which was obtained from a tiger with contact to a COVID‐19 infected zookeeper,^63^ have also been used. Both contain the D614G S‐protein mutation, which has been suggested to alter SARS‐CoV‐2 fitness,^94^, ^95^ promote viral transmission,^91^ and transduce cells more effectively in vitro.^96^ To the author's knowledge, no other variants have been assessed.

## 10 SUMMARY

The SARS‐CoV‐2 pandemic is now in its third year. While the number of seriously ill people is decreasing, there is still an alarmingly high number of people being infected every single day, allowing the virus to continue to move rapidly from person to person and providing the virus with ample opportunities for further mutations. The number of organ recipients has also continued to increase during the pandemic, and solutions to resolve the lack of available organs from human donors are still needed. Pigs are ideal for xenotransplantation and much needed pig‐to‐human organ transplantation is evolving quickly. However, pigs can harbor both pig‐ and human‐specific viruses which may pose a risk for the human organ recipient. It appears that SARS‐CoV‐2 infection from pigs is currently not a major threat, but this may change in the future should variants become more adapted to pigs. Because of this, experts should periodically assess the situation to make sure that this has not occurred. In particular, studies of whether pigs are more susceptible to infection with some of the newer SARS‐CoV‐2 variants, including any new variants of concern, is urgently warranted. In addition, transplant organ recipients in general should be cautious of other zoonotic SARS‐CoV‐2 sources including pets.
